# Expanding Selection Criteria to Repairable Diseased Hearts to Meet the Demand of Shortage of Donors in Heart Transplantation

**DOI:** 10.7759/cureus.25485

**Published:** 2022-05-30

**Authors:** John H Yazji, Pankaj Garg, Ishaq Wadiwala, Mohammad Alomari, Emad Alamouti-Fard, Md Walid Akram Hussain, Samuel Jacob

**Affiliations:** 1 Cardiothoracic Surgery, Mayo Clinic, Jacksonville, USA

**Keywords:** prioritization, repairable hearts, organ donor shortage, marginal hearts, donor heart, selection criteria, heart transplant

## Abstract

Heart transplant surgery is considered the destination therapy for end-stage heart disease. Unfortunately, many patients in the United States of America who are eligible candidates for transplants cannot undergo surgery due to donor shortage. In addition, some donors' hearts are being labeled as unacceptable for transplant surgery because of the rigorous and restricted rules placed on the approval process of using a donor's heart. Over the last few decades, the rising discrepancy between the scarcity of donor hearts and the demand for such organs has led to the discussion of expanding the donor heart selection criteria. A softer view on using marginal hearts for transplants would help those on the waitlist to receive a heart transplant. Marginal hearts that contain the hepatitis c virus (HCV), COVID-19, older age, or repairable heart defects have become viable options to use for a heart transplant. Also, the prioritization based on the new heart allocation system would help efficiently decide which recipients would be the first to get a donor's heart. Recently there has been a consensus to broaden the eligibility of donor's hearts by accepting valvular abnormalities, coronary artery disease, and congenital abnormalities. This review highlights some of those expansions in selection criteria in particular using repairable hearts, which could be fixed in the operating room on the back table before transplantation.

## Introduction and background

In the United States of America, less than 50% of the potential organ donors do, in fact, become actual donors. The field of medicine has evolved as better medicine and technology have become available [1]. With these new developments over the years, the selection criteria for a patient receiving and donating a heart are under immense pressure to be changed and updated. Many hearts are being left out for not being able to meet the specific requirements needed to be considered a good donor heart. Expanding the selection criteria to use a donor's heart would alleviate some of the stress on the waitlist pool.

The process of getting a transplant candidate approved for receiving a heart donation is exhaustive. The transplant centers begin the process by placing the patient on the transplant waitlist. Once placed on the waitlist, the potential heart donor matching process starts by identifying and surveying different candidates. The survey lists information about the donor, such as confirmation of brain death, consent for donation, blood type, etc. When a donor's heart has been matched, a transplant surgeon is sent to the location of the donor's heart for final inspection and approval to be used for transplant. Finally, once the donor's heart has been accepted, it is brought to the recipient facility to be implanted [2].

**Figure 1:**
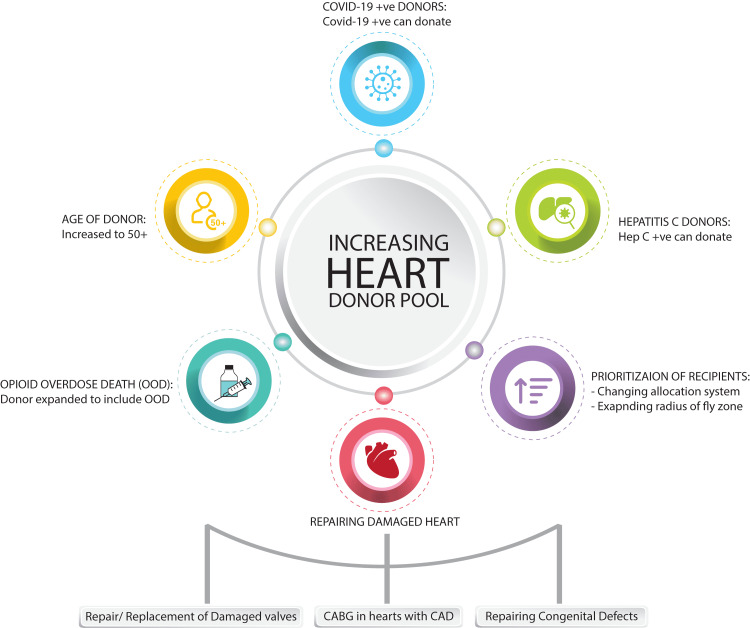
Different methods of expanding donor pool criteria for heart transplant Some of the expanding criteria for donor pool expansion, including repairable hearts, which needs coronary artery bypass grafting (CABG) for coronary artery disease (CAD), valve repairs or replacement, and congenital heart defects repaired.

Since the list of the waitlist has been growing at a rapid rate, there has been greater pressure of expanding the selection criteria. With the scarcity of donor hearts available, there has been an urgency to use marginal hearts. Marginal hearts have problems that would not generally be acceptable under current United Network for Organ Sharing (UNOS) guidelines [3]. With greater evidence and research, expanding the selection criteria by using marginal hearts for cardiac transplantation has been talked about as a possibility to battle the shortage of hearts available (Figure 1).

## Review

Repairable hearts

For a long time, a donor heart that had the valvular disease or needs valve surgery was disqualified for a heart transplant. Any donor heart with echocardiographic evidence of valvular disease abnormality is normally considered a contraindication to donation in the United States [4]. Recently, there has been a consensus to broaden the eligibility of donor hearts by accepting hearts with valvular abnormalities [5]. Having a mitral and aortic valve replacement on the donor's heart during heart transplantation is a solution for using repairable donor hearts. There is much risk involved when performing valve replacement during heart transplantation because there is a concern for durability and viability during/after the repair. Also, the extra ischemic time needed to perform a repair or replacement surgery must be carefully considered before going into repairable heart transplantation [6]. A case study by Patel et al. described a 71-year-old male patient with advanced heart failure who was placed on the waitlist as he was not a candidate for revascularization or myocardial ablation. The patient waited for 51 days for a donor heart to be selected. The donor's heart came from a 17-year-old boy who had a moderate aortic valve regurgitation. Before the orthoptic heart transplantation, a back-table aortic valve replacement was performed on the donor's heart. The patient recovered from surgery well and was discharged from the hospital after 11 days. At the one- and two-year follow-ups, the echocardiograms showed a well-functioning bioprosthetic aortic valve [7]. This case demonstrated that a donor heart with an aortic valve defect but otherwise healthy can be used in excellent function for heart transplantation. For patients listed on UNOS, the one-year mortality rate is between 8% and 14% for those who do not receive a transplant [8]. The strict criteria that identify a heart for transplant have led to the shortage of organ donation. Even though having aortic valve replacement is feasible for transplantation, there are still a few limitations to this approach. Replacing an aortic valve on a donor's heart before heart transplantation leads to prolonged surgical and ischemia time. Also, there is a possibility of the surgically repaired valve failure requiring more valve replacement redo surgeries in the future [9]. Nevertheless, using a donor's heart that needs to be repaired can be a viable option for relaxing the criteria for using these types of donor hearts, increasing the donor pool, and decreasing the amount of time patients are on the waitlist [10].

Heart failure is a major global health issue, affecting approximately 5.8 million people in the United States and over 23 million people worldwide. Heart failure is a leading cause of hospitalizations among individuals aged 65 and older, with a mortality rate of over 10% every year. As a result, heart failure consumes a significant amount of healthcare resources, putting immense pressure on the healthcare system [11-13]. Having cardiac transplantation for those patients dealing with heart failure has become a routine procedure to perform. Unfortunately, due to the shortage of donor heart availability many patients around the world are unable to receive an organ. A case report by Saito et al. described a 21-year-old woman who underwent a left ventricular (LV) assist system device as she was waiting for a heart transplant. The patient underwent a heart transplant after 853 days on the waitlist. The donor's heart was from a 30-year-old man that had mild LV hypertrophy, bicuspid aortic valve, and mild aortic stenosis. The donor's heart required a bench replacement of the aortic valve in which a mechanical vale was used. The recipient did not have any complications post-transplantation and was doing well until eight months post-surgery [14]. Hopefully, with case reports like the 21-year-old female recipient, the variables that previously rendered a heart unsuitable for transplantation are changing. One of the main issues with using a mechanical valve for a donor's heart is the requirement of warfarin after transplantation. Using warfarin can put the patient at a higher risk of bleeding, which is potentially dangerous for all patients but especially those who had heart transplantation [15]. Clinical studies are underway on the possibilities of using alternatives to warfarin for patients with a mechanical heart valve. Another issue is the risk of structural valve degeneration of the aortic bioprosthesis is higher in younger patients than in older patients. Heart transplant recipients in their 20s have a 10-year survival rate of more than 50%, which is improving every year, but there is a substantial risk of reoperation in 15 years [16,17].

A repairable heart transplant case by Sultan et al. reports on a 53-year-old male who had ischemic cardiomyopathy. The recipient received a heart of a 37-year-old donor who had a bicuspid valve with severe aortic insufficiency. A concomitant aortic valve replacement was performed on the donor's heart. The recipient is doing well at the six-month and one-year follow-ups, and CT scans showed no aortic dilation [18]. As the heart transplant waiting list continues to lengthen, the shortage of heart organ donors has demanded that the selection criteria be expanded. Using marginal hearts for recipients who are critically ill or have been on the waiting list for an extended period, will help to shrink the gap between supply and demand for heart organs. A case report by Rao et al. described a situation when a 65-year-old male who had been on the waitlist for over two years received a donor heart from a 58-year-old male. The donor's heart had two-vessel CAD and a bicuspid valve which needed aortic valve replacement and CABG. With the recipient having critical heart failure and a declining condition, an orthotopic heart transplant with concurrent double coronary bypass graft was performed. The postoperative recovery went well, and the recipient did well until 16 months after surgery [19]. This case was complex as it evolved using a CABG on a donor heart that was above the age of 50. With the recipient in good condition, this exhibits that using marginal hearts can be a viable option for people who are in dire need of transplant surgery.

**Figure 2:**
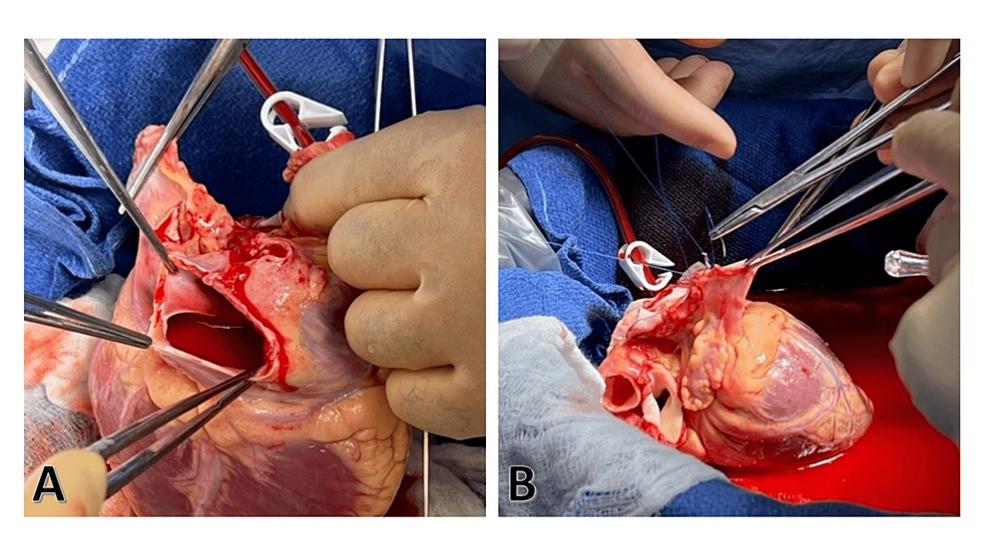
Removal of duplicate LSVC (A) A heart with congenital left superior vena cava. (B) Repairing the anomaly on the back table before orthotopic heart transplantation. LSVC - left superior vena cava

In our institution, we have used hearts with congenital anomalies, such as persistent total left superior vena cava (LSVC) The heart was repaired on the back table and transplanted successfully (Figures 2A, 2B). Before accepting hearts with congenital anomalies, other co-existing anomalies should also be excluded, such as coronary sinus orifice atresia, bicuspid aortic valve, conduction system anomalies, and atrial septal defect.

Even though using a repairable can be a great option for expanding the donor selection pool, there are a few limitations. When using a repairable heart for a heart transplant, the repairable heart must first be operated on before placing inside the recipient. Repairing the donor's heart will lead to a longer ischemic time of the donor's heart and an increase in the primary graft dysfunction, which will increase the overall operative time [20-23]. Also, using a repairable heart will escalate the cost of patient care. After the heart transplant, the patient might need additional surgery in the future.

Hepatitis C virus 

Two decades back, hepatitis C virus (HCV) infection was considered highly fatal due to its association with hepatocellular carcinoma and lack of any effective treatment [24]. The trouble with using an HCV-positive heart was that the recipients of an HCV-positive heart had become HCV-positive themselves, which led to increased mortality and poor long-term outcome [25,26]. Therefore, the presence of HCV was considered an absolute contraindication for organ donation. However, with the availability of direct-acting antiviral drugs that have a high-cure rate of HCV [27], it is now possible to cure or effectively control the disease. Direct-acting antivirals work by impeding viral replication because they target specific nonstructural viral proteins [28]. Direct-acting antiviral drugs such as Sofosbuvir has been used in the last few years particularly, and its positive results have made using HCV hearts a possibility [29]. A study done by Dharmavan et al. looked at donor trends for HCV-positive hearts in the past 20 years and saw a significant increase in the survival rates of patients receiving HCV positive. The 30-day and one-year survival rates of recipients with HCV-positive hearts have drastically improved over the last couple of years with the help of direct-acting antivirals [30]. Recipients have a high chance, at least 10%, of dying while on the waiting list, which continues to grow the more time spent on the waitlist. Due to clinical worsening, over 5% of waitlisted patients are removed from the list before heart transplantation [31,32]. For those patients still on the waitlist, there is a possibility of having comorbidities such as renal dysfunction, stroke, or being placed on mechanical circulatory support [33]. Even though this possibility is in the early stages of development, using HCV-positive hearts will significantly supplement the amount of heart donor organs available, reducing the amount of time spent on the waitlist.

Age

Another way to broaden the donor pool is by accepting the hearts of older patients. Even though there is no age limit to be a candidate for a heart organ donation, it is generally less desirable to receive a donor heart above the age of 50 years [34]. Donor hearts in this age group have higher risk factors such as heart failure and CAD. Blanche et al. did a study on having heart transplantation with donors above the age of 50 years. The study exhibited that patients could have a good long-term survival rate with minimal risk factors and similar outcomes compared to patients who received a donor heart from a younger patient. Using older hearts gives for a larger donor pool while also allowing for more efficient heart selection without compromising long-term results [35]. There is a matter of concern for older patients (≥60 years old) receiving a heart above the age of 50 years. When a cardiac transplant is performed on those types of patients, there is a lower chance of survival as there is an increase in mortality and in-hospital deaths. Also, recipients who have received a donor heart above the age of 50 years combined with longer ischemia times have led to poor outcomes. It was found that if the ischemia time lasted more than three to four hours then the recipient could have early mortality [36-39]. Decisions of doing a heart transplant must be made between the patient and the doctor regarding whether or not to go ahead with the transplantation [40].

Prioritization

Recently, a new heart allocation policy was enacted with the purpose of giving new hearts to patients who are the sickest in order to decrease the amount of time on the waitlist [41]. The previous allocation system was a three-tier system labeled 1A, 1B, and 2. The three-tier system had some major disadvantages as it was very ambiguous for the patients who needed a donor heart the most and prolonged the time on the waitlist [42]. The new allocation policy has a six-tiered system that ranges from 1 to 6, with one being the highest priority and six being the lowest priority. An example of the difference between the two policies is in the old heart allocation policy, a patient with venoarterial extracorporeal membrane oxygenation and a stable patient on LV assist device (LVAD) support would be in the same tier. In contrast, in the new heart allocation policy, the patients would be in status 1 and 4, respectively [43]. A recent study by Liu et al. demonstrated that using the new heart allocation system there was a significant decrease in patient support by LVADs and more likely to be supported by intra-aortic balloon pumps. Patients resulted having decreased time on the waitlist, but an increased inpatient hospital length before the transplant occurred [44].

Opioid overdose death

Due to the worsening of the opioid epidemic, there has been a rise in opioid overdose death donors (OODDs). Sadly, most of the patients who contribute to the OODDs are younger than the age of 45 years and greatly contribute to the donor pool [45]. According to the Centers for Disease Control and Prevention, in 2020, there were over 90,000 deaths related to opioid overdose [46]. It is worrisome to use hearts from OODDs as there might be cardiac complications associated with the patient using opioids. One study reported that in patients who have been hospitalized with heart failure, about 25% of them are related to the use of opioids as prescription [47]. Opioid users can have various issues such as cardiovascular, respiratory, and neurological problems [48]. Patients who have received other donor organs, for example, liver and kidney, have higher rates of death and graft failures [49]. A long-term study completed by Baran et al. saw no significant difference in survival between recipients who received a donor heart with a history of drug abuse compared to those who do not [50]. A more lenient view of OODDs hearts will help further to expand the donor pool.

COVID-19

Heart organ donation and transplantation were significantly affected in the United States due to the COVID-19 pandemic. Recipients on the waitlist had an increased risk of acquiring COVID‐19 [51]. From March 2020 to May 2020, organ authorization and recovery rates fell by 11% and 17%, respectively, as social distancing reduced hospital admissions and traumatic deaths [52]. There was a concern for the risk factors that can occur not only to the recipient of a positive COVID-19 donor but also to the donor heart retrieving team. In the early months of the pandemic, it was universally accepted that a patient who had a history of or currently has the COVID-19 infection would not be able to donate any organs. But with the rising number of deceased donors due to COVID-19 and its complications, using donor patients who had or have SARS-CoV-2 infection became a possible thought. Millions of people in the United States have recovered from coronavirus infections; unfortunately, some of these people will die due to unrelated conditions and become organ and tissue donors. A case series by Neidlinger et al. indicated that using the heart of a COVID-19 patient did not transmit the infection to the cardiac recipient as well as to the retrieval team [53]. Even with the limited research, the case series showed that using the heart of a COVID-19 donor could be plausible, especially for those who died discrete from COVID-19. Before automatically disqualifying organs from donors with a history of COVID-19, the transplantation communities should carefully consider the risks and benefits of using the hearts.

## Conclusions

Due to the relative lack of supply of donor hearts and the growing number of patients on the waiting list, cardiologists and surgeons have become more enlightened in their donor heart selection. There is substantial evidence that using marginal donors produces satisfactory results, making it justifiable to address the growing donor organ shortage. With the increasing need for heart transplant surgery, especially for those who are older and have severe medical conditions, recipients may benefit from donors with marginal hearts who have been properly examined. The purpose of expanding the selection criteria is to broaden the range of patients' hearts that can be used successfully and increase the number of hearts in the donor pool. With the continuous advancement in medicine, ways to combat the need for a heart transplant and the shortage of heart donors will continue to be created. Further studies are needed to discuss the possible risk factors using the methods discussed to expand the selection criteria.
